# Gestational diabetes among women of migrant origin in Finland—a population-based study

**DOI:** 10.1093/eurpub/ckab078

**Published:** 2021-05-31

**Authors:** Kalpana Bastola, Päivikki Koponen, Natalia Skogberg, Mika Gissler, Tarja I Kinnunen

**Affiliations:** 1 Unit of Health Sciences, Faculty of Social Sciences, Tampere University, Tampere, Finland; 2 Department of Health, Finnish Institute for Health and Welfare, Helsinki, Finland; 3 Department of Information Services, Finnish Institute for Health and Welfare, Helsinki, Finland; 4 Division of Family Medicine, Department of Neurobiology, Care Sciences and Society, Karolinska Institute, Stockholm, Sweden

## Abstract

**Background:**

Migrant women may have a higher risk for gestational diabetes mellitus (GDM) and the related adverse outcomes. We studied the prevalence of GDM among migrant-origin women in Finland.

**Methods:**

This study used data from the nationwide Medical Birth Register. Information on the most recent singleton births of women delivering between 2004 and 2014 (*N* = 379 634) was included. Women were classified into nine regional categories based on the country of origin. Finnish origin women were the reference group. Generalized linear models adjusted for maternal age, parity, socioeconomic position, pre-pregnancy body mass index and year of delivery were used to study the association between region/country of origin and GDM.

**Results:**

Among the study population, almost 8% were of migrant origin. The prevalence of GDM varied from 6.1% (women of Latin American/Caribbean origin) to 18.4% (South Asian origin), compared to 8.7% in the Finnish reference group. When adjusted for confounders, women of South Asian, East Asian, Middle Eastern/North African and Russian/former USSR origin had a higher risk for GDM than Finnish origin women. By country of origin, women originating from Pakistan, Bangladesh, Sri Lanka, India, Afghanistan, Nepal, China, Philippines, Vietnam, Thailand, Morocco, Turkey, Iran, Iraq and former USSR had a higher risk for GDM than Finnish origin women.

**Conclusions:**

There is substantial variation in the prevalence of GDM by country of origin. Women of South Asian, East Asian and Middle Eastern/North African origin had the highest risk for GDM and may warrant special attention.

## Introduction

Gestational diabetes mellitus (GDM) is the presence of a higher blood glucose level during pregnancy.^1^ The prevalence of GDM varies between 5% and 16% globally, depending upon the population, screening strategy and diagnostic criteria.^2^ GDM usually resolves following delivery but it may have wide-ranging health consequences for the mother and the newborn. Previous evidence suggests that GDM increases the risk of additional pregnancy complications, such as preeclampsia and preterm birth.^1^ Approximately 60% of women with a history of GDM develop type 2 diabetes mellitus (T2DM) later in life.^3^ Furthermore, the risk of a cardiovascular event is increased for women with a history of GDM.^4^ Newborns of women with GDM are often macrosomic due to fetal overgrowth. Later in life, children of a mother with GDM have an increased risk for obesity, T2DM, cardiovascular diseases and associated metabolic diseases.^5^^,^^6^ Females born to a mother who had GDM during her pregnancy are more likely to have GDM in their pregnancies.^7^ This contributes to a vicious intergenerational cycle of obesity and diabetes that impacts the health of the population as a whole.

There are several well-established risk factors for GDM. These include maternal overweight and obesity,^8^ advanced maternal age,^9^ and family and personal history of GDM.^10^ Furthermore, excessive gestational weight gain during pregnancy,^11^ dietary habits such as a diet high in saturated fat, processed foods and refined sugar,^12^ low- or high-birth weight of newborns in a previous pregnancy,^13^ and specific diseases of insulin resistance^14^ are also associated with the increased risk of GDM. Many of these risk factors are either directly or indirectly associated with impaired β-cell function and/or insulin resistance.^15^ Sympathetic nervous overactivity is also involved in the pathogenesis of insulin resistance and contributes to development of GDM by influencing adiposity and glucose utilization.^15^

Ethnicity or the country of origin has been identified as one of the risk factors for GDM.^16–19^ In many studies, ethnicity and migration are used interchangeably although they are not identical concepts. While some ethnic differences may be explained by genes and cultural practices, others may be explained by migration-related reasons such as poverty in the country of origin before migration and rapid change in lifestyle after migration. Studies from the USA and Australia have found that women of Hispanic, African, Native American, South or East Asian, Pacific Islands or Indigenous Australian origin are at a higher risk for developing GDM compared to the general population in these countries.^16^^,^^17^ Two recent studies found that those women who were born in South Asia, East Asia, Middle East and North Africa had higher odds for GDM as compared to women who were born in Norway and Denmark, respectively.^18^^,^^19^

There is very little information on the prevalence of GDM in migrant or ethnic minority populations in Finland. One previous study found that women of Kurdish origin had a higher risk of GDM compared to the general Finnish population.^20^ The study was based on a relatively small sample of three migrant groups and could not be generalized to all women of migrant origin in Finland.^20^ In recent years, the national prevalence of GDM increased from 11.5% in 2015 to 19% in 2019 in Finland.^21^ Most of the recent population growth in Finland has been due to migration flows and the fact that migrant origin women had a higher birth rate than Finnish origin women.^22^ Therefore, it is essential to identify the high-risk groups for GDM among migrant origin women. In this study, we compared the prevalence of GDM by region of origin and identified the groups at the highest risk for GDM among all women giving birth in Finland between the years 2004 and 2014.

## Methods

### Study population

This study utilized the data from the national Medical Birth Register (MBR) of Finland. The MBR collects data on the mother’s sociodemographic characteristics, previous pregnancies and deliveries, present pregnancy and its monitoring, delivery and complications, and information on the newborn health.^23^ The present study included information on each woman's most recent pregnancy in Finland between January 2004 and December 2014 (*n* = 389 758). After excluding multiple births (*n* = 7525) and women with preexisting diabetes (International Statistical Classification of Diseases and Related Health Problems ICD-10 codes: O24.0–O24.3, *n* = 2 395), the final study sample was 379 634. Data on the country of origin and socioeconomic position were obtained from national statistical authority Statistics Finland and linked to study data by using the personal identification code for each woman.

The migrant groups were categorized *a priori* based on the United Nations’ geographical classification of countries. Country of origin was defined as the country of birth of the pregnant woman's parents. If both parents were born abroad, the country of birth of the woman's biological mother was considered to be the primary country of origin. If at least one of the parents was born in Finland, the country of origin was defined as Finland. Women were classified into nine regional categories according to their country of origin: (i) Finland; (ii) Western Europe/North America/Oceania (i.e. Western Europe); (iii) Eastern Europe; (iv) Russia and the former Union of Soviet Socialist Republics (USSR); (v) South Asia; (vi) East Asia; (vii) Sub-Saharan Africa; (viii) Middle East/North Africa; and (ix) Latin America/Caribbean. Women with an unknown country of origin (*n* = 231, 0.06%) were excluded. A list of countries and numbers of women in each group are presented in Supplementary table S1.

### Outcome

The presence of GDM was extracted from the MBR based on the ICD-10 codes. We included ICD-10 codes O24.4 (gestational diabetes), O24.9 (unspecified gestational diabetes in pregnancy), and P70.0 or P70.1 (child of a diabetic mother) to identify the presence of GDM. All these ICD-10 codes were compiled together and finally dichotomized (yes/no).

In Finland, GDM is diagnosed when fasting plasma glucose value is ≥5.3 mmol/l, the 1-h value is ≥10.0 mmol/l or the 2-h value is ≥ 8.6 mmol/l.^24^ Oral glucose tolerance testing is recommended for all pregnant women, except for those who are categorized to be at lower risk, that is primiparae women who are less than 25 years old, normal weight (18.5–24.9 kg/m^2^), and do not have a family history of T2DM.^24^ The screening of GDM changed slightly in 2008. Before 2008, GDM test was recommended for the high-risk groups only.^25^

### Background characteristics

Mother's age at delivery was categorized into four categories: <25, 25–29, 30–34, and ≥35 years. Socioeconomic position was categorized into five categories: upper-level employees (administrative, managerial, professional, and related occupations), lower-level employees (administrative and clerical occupations), manual workers, other (including pensioners/homemakers/students), and unknown. Parity (number of previous births) was categorized as 0, 1, and ≥2. Pre-pregnancy BMI was categorized as underweight (<18.5 kg/m^2^), normal weight (18.5–24.9 kg/m^2^), overweight (25–29.9 kg/m^2^) and obese (≥30 kg/m^2^). The delivery year was categorized as 2004–2007, 2008–2011 and 2012–2014 and used in adjustments to account for changes in the screening strategy for GDM in Finland from 2008 onwards and changes in the proportion of deliveries of migrant women of all deliveries during the study period.

### Statistical analyses

Descriptive data were reported as numbers of observations and prevalence (%). Generalized linear models with the log-link function were used to obtain relative risk (RR) estimates with 95% confidence intervals (CIs). Finnish origin women were the reference group. Model I was an unadjusted model whereas Model II was adjusted for age, socioeconomic position, pre-pregnancy BMI, parity, and delivery year. Age, BMI, parity and delivery year were used as continuous variables in the adjusted model. Furthermore, after identifying the migrant groups at a higher risk for GDM, we run respective, additional analyses for GDM risk by individual countries among the high-risk migrant groups, using Finnish origin women as the reference category. In the individual country analyses, countries with more than 10 GDM cases were included to ensure statistical power and data protection. All analyses were performed in Statistical Package for the Social Sciences (SPSS, version 23; SPSS Inc.).

### Ethical approval

The permission to use the data was obtained from the respective registries from the Finnish Institute of Health and Welfare (THL) and Statistics Finland. We analyzed and stored the anonymized data at THL, following THL’s data safety regulations.

## Results

In our study population, almost 8% (*n* = 31 321) of women were of migrant origin. Very few women (*n* = 216, 0.01%) of all women were second-generation women of migrant origin. Women of Russian/former USSR origin constituted the largest (*n* = 11 961), whereas women of Latin American/Caribbean origin (*n* = 736) were the smallest migrant origin group (table 1).

**Table 1:** Background characteristics by region of origin during the most recent delivery, all singleton births, 2004–2014, (number and crude percentage)

Variables	Finland, (*n* = 348 313)	Western Europe^a^ (*n* = 2276)	Eastern Europe (*n* = 2560)	Russia, former USSR (*n* = 11 961)	South Asia, (*n* = 1893) Number (%)	East Asia, (*n* = 4933)	Sub-Saharan Africa (*n* = 3522)	Middle East/North Africa (*n* = 3440)	Latin America, Caribbean (*n* = 736)
Age at delivery (years)									
<25	44 419 (12.8)	160 (7.0)	491 (19.2)	1919 (16.0)	354 (18.7)	538 (10.9)	742 (21.1)	680 (19.8)	73 (9.9)
25–29	92 580 (26.6)	444 (19.5)	799 (31.2)	3654 (30.5)	725 (38.3)	1333 (27.0)	976 (27.7)	1004 (29.2)	167 (22.7)
30–34	121 793 (35.0)	863 (37.9)	768 (30.0)	3606 (30.1)	581 (30.7)	1698 (34.4)	1005 (28.5)	924 (26.9)	253 (34.4)
>35	89 521 (25.7)	809 (35.5)	502 (19.6)	2782 (23.3)	233 (12.3)	1364 (27.7)	799 (22.7)	832 (24.2)	243 (33.0)
Socioeconomic position									
Upper level employees	71 874 (20.6)	768 (33.7)	298 (11.6)	1249 (10.4)	312 (16.5)	714 (14.5)	137 (3.9)	156 (4.5)	175 (23.8)
Lower level employees	136 035 (39.1)	550(24.2)	439 (17.1)	2510 (21.0)	243 (12.8)	777 (15.7)	435 (12.4)	289 (8.4)	158 (21.5)
Manual workers	77 393 (22.2)	436 (19.2)	882 (34.5)	4183 (35.0)	441 (23.3)	1811 (36.7)	681 (19.3)	882 (25.6)	160 (21.7)
Others^b^	42 848 (12.3)	266 (11.7)	558 (21.8)	2381 (19.9)	578 (30.5)	1064 (21.6)	1128 (32.0)	1140 (33.1)	163 (22.1)
Unknown	20 163 (5.8)	256 (11.2)	383 (15.0)	1638 (13.7)	319 (16.9)	569 (11.5)	1141 (32.4)	973 (28.3)	80 (10.9)
Previous births									
None	104 082 (29.9)	883 (38.9)	831 (32.5)	4318 (36.1)	810 (42.9)	2000 (40.6)	878 (24.9)	952 (27.7)	327 (44.4)
One	141 477 (40.6)	861 (37.9)	999 (39.0)	4979 (41.6)	665 (35.2)	1876 (38.0)	909 (25.8)	1180 (34.3)	275 (37.4)
Two or more	102 590 (29.5)	527 (23.2)	730 (28.5)	2658 (22.2)	414 (21.9)	1055 (21.4)	1733 (49.2)	1305 (38.0)	134 (18.2)
Pre-pregnancy body mass index^c^									
Underweight	10 354 (3.1)	111 (5.2)	146 (6.0)	856 (7.5)	106 (5.9)	673 (14.4)	143 (4.3)	106 (3.3)	30 (4.4)
Normal weight	202 533 (60.9)	1426 (66.8)	1554 (64.0)	7711 (67.9)	1043 (58.1)	3400 (73.0)	1424 (43.3)	1554 (48.3)	487 (71.4)
Overweight	75 720 (22.8)	379 (17.9)	529 (21.8)	1941 (17.1)	496 (27.6)	494 (10.6)	1048 (31.9)	1075 (33.4)	121 (17.7)
Obese	44 206 (13.3)	213 (10.1)	200 (8.2)	853 (7.5)	150 (8.4)	91 (2.0)	675 (20.5)	482 (15.0)	44 (6.5)
Year of delivery									
2004–7	94 709 (27.2)	574 (25.2)	456 (17.8)	2590 (21.7)	286 (15.1)	997 (20.2)	440 (12.5)	626(18.2)	160 (21.7)
2008–11	117 066 (33.6)	769 (33.8)	832 (32.5)	4050 (33.9)	586 (31.0)	1741 (35.3)	980 (27.8)	1143 (33.2)	272 (37.0)
2012–14	136 538 (39.2)	933 (41.0)	1272 (49.7)	5321 (44.5)	1021 (53.9)	2194 (44.5)	2102 (59.7)	1671 (48.6)	304 (41.3)

a Western Europe, North America and Oceania.

b Others include students, housewives, unemployed and pensioners.

c Missing values for pre-pregnancy body mass index in each group from the left to the right were 4.5%, 6.9%, 5.1%, 5.0%, 5.8%, 5.6%, 6.6%, 6.5% and 7.3%, respectively. Missing values for parity were <1% in each category, no missing values for the age at delivery, socioeconomic position and year of delivery.

Compared with Finnish women, the percentages of women in the upper and lower level employees’ categories were lower for all other migrant origin women except for women in the Western Europe group. The socioeconomic position of 61-64% of women from the Middle East/North Africa and Sub-Saharan African origin was categorized as others (students, housewives, unemployed and pensioners) or unknown. Women from Sub-Saharan African and Middle Eastern/North African origin had the higher percentages of two or more parity than any other group. Sub-Saharan African and Middle Eastern/North African women had a higher, whereas and East Asian women had a lower prevalence of overweight and obesity compared with Finnish origin women.

The prevalence of GDM was the highest among women of South Asian origin (*n* = 349, 18.4%) and lowest among women of Latin American/Caribbean origin (*n* = 45, 6.1%) (table 2). The prevalence of GDM was 8.7% (*n* = 30 454) among Finnish origin women. In addition, table 2 presents the unadjusted and adjusted risk ratios (RRs) and 95% CIs for the risk of GDM in each migrant group compared with the Finnish origin group. The unadjusted results showed that women of Western European (RR 0.84; 95% CI 0.72–0.99), and Latin American/Caribbean origin (RR 0.68 95% CI 0.50–0.92) had a lower risk for GDM compared with the Finnish origin women. A higher risk of GDM was observed among women of South Asian (RR 2.36; 95% CI 2.10–2.66), East Asian (RR 1.29; 95% CI 1.16–1.43), Sub-Saharan African (RR 1.29; 95% CI 1.16–1.43) and Middle Eastern/North African origin (RR 1.53; 95% CI 1.38–1.67) compared with Finnish origin women. When adjusting for confounders, the RRs increased among women of South Asian (RR 2.95; 95% CI 2.60–3.34) and East Asian (RR 2.12; 95% CI 1.93–2.33) origin, decreased slightly among women of Middle Eastern/North African origin (RR 1.36; 95% CI 1.22–1.51), and increased slightly, becoming statistically significantly higher among women of Russian/former USSR origin (RR 1.17; 95% CI 1.10–1.26) compared with Finnish origin women. However, the risk of GDM did not differ between women originating from Western Europe, Sub-Saharan African or Latin America/Caribbean and the women of Finnish origin anymore after adjusting for confounders.

**Table 2:** Risk ratio (RR) and 95% confidence interval (CI) for having gestational diabetes mellitus during the most recent pregnancy by region of origin

Regions	GDM *n* (%)	Model I^b^ RR (95% CI)	Model II^c^ RR (95% CI)
Finland	30 454 (8.7)	Reference	Reference
Western Europe^a^	170 (7.5)	0.84 (0.72–0.99)	0.89 (0.75–1.05)
Eastern Europe	215 (8.4)	0.96 (0.83–1.10)	1.11 (0.96–1.29)
Russia/Former USSR	990 (8.3)	0.94 (0.88–1.01)	1.18 (1.10–1.27)
South Asia	349 (18.4)	2.36 (2.10–2.66)	2.95 (2.60–3.34)
East Asia	542 (11.0)	1.29 (1.18–1.41)	2.12 (1.93–2.33)
Sub Saharan Africa	386 (11.0)	1.29 (1.16–1.43)	0.91 (0.81–1.02)
Middle East/North Africa	439 (12.8)	1.53 (1.38–1.67)	1.36 (1.22–1.51)
Latin America/Caribbean	45 (6.1)	0.68 (0.50–0.92)	0.81 (0.59–1.18)

a Western Europe, North America and Oceania.

b Unadjusted generalized linear model.

c Generalized linear model adjusted for age, socioeconomic position, pre-pregnancy body mass index, parity and year of delivery.

Table 3 shows results from the analyses by individual country of origin among the high-risk groups. The adjusted model showed that among women of South Asian origin, women from Pakistan, Bangladesh, Sri Lanka, India, Afghanistan and Nepal had a higher risk for GDM compared with Finnish origin women. Similarly, among women of East Asian origin, women from China, Philippines, Vietnam and Thailand had a higher risk for GDM than Finnish women. Furthermore, among women of Middle Eastern/North African origin, women from Morocco, Turkey, Iran and Iraq had a higher risk for GDM compared with Finnish origin women. Finally, among women of Russia/former USSR origin, women originating from former the USSR had a higher risk for GDM and women from Estonia, Russia and Latvia did not differ from Finnish origin women.

**Table 3:** Risk ratio (RR) and 95% confidence interval (CI) for having gestational diabetes mellitus during the most recent pregnancy by country of origin for the highest risk groups

Countries^a^	Total women	GDM *n* (%)	Model I^b^ RR (95% CI)	Model II^c^ RR (95% CI)
Finland	348 313	30 454 (8.7)	Reference	Reference
Russia/Former USSR	11 961	990 (8.3)	0.94 (0.88–1.01)	1.18 (1.10–1.27)
Estonia	3504	289 (8.2)	0.94 (0.83–1.06)	1.08 (0.95–1.23)
Former USSR	7136	597 (8.4)	0.95 (0.88–1.04)	1.25 (1.14–1.36)
Russia	682	56 (8.2)	0.93 (0.71–1.23)	1.20 (0.89–1.61)
Latvia	200	20 (10.0)	1.16 (0.73–1.84)	1.41 (0.86–2.28)
South Asia	1893	349 (18.4)	2.36 (2.10–2.66)	2.95 (2.60–3.34)
Afghanistan	528	89 (16.9)	2.12 (1.68–2.66)	2.34 (1.83–3.00)
Bangladesh	257	57 (22.2)	2.98 (2.22–3.99)	3.73 (2.74–5.09)
India	623	104 (16.7)	2.09 (1.69–2.58)	2.88 (2.31–3.59)
Nepal	115	12 (10.4)	1.22 (0.67–2.21)	1.92 (1.04–3.54)
Pakistan	235	58 (24.7)	3.42 (2.54–4.60)	4.17 (3.02–5.77)
Sri Lanka	134	29 (21.6)	2.88 (1.91–4.35)	3.33 (2.14–5.18)
East Asia	4933	542 (11.0)	1.29 (1.18–1.41)	2.12 (1.93–2.33)
Thailand	1661	181 (10.9)	1.28 (1.09–1.49)	2.03 (1.72–2.38)
Vietnam	960	88 (9.2)	1.05 (0.85–1.31)	1.95 (1.55–2.46)
Myanmar	184	19 (10.3)	1.20 (0.75–1.93)	1.67 (0.99–2.80)
Philippines	477	63 (13.2)	1.59 (1.22–2.07)	2.40 (1.81–3.16)
Japan	230	17 (7.4)	0.83 (0.51–1.37)	1.34 (0.81–2.21)
China	1131	152 (13.4)	1.62 (1.37–1.92)	2.79 (2.33–3.33)
Middle East/North Africa	3440	439 (12.8)	1.53 (1.38–1.67)	1.36 (1.22–1.51)
Turkey	755	94 (12.5)	1.48 (1.20–1.84)	1.51 (1.20–1.89)
Tunisia	70	12 (17.1)	2.16 (1.16–4.02)	1.85 (0.95–3.62)
Sudan	185	16 (8.6)	0.99 (0.59–1.65)	0.70 (0.40–1.20)
Morocco	334	47 (14.1)	1.71 (1.26–2.33)	1.65 (1.19–2.27)
Iran	554	69 (12.5)	1.49 (1.15–1.91)	1.47 (1.12–1.93)
Iraq	1264	163 (12.9)	1.55 (1.31–1.82)	1.20 (1.00–1.44)
Algeria	98	15 (15.3)	1.89 (1.09–3.27)	1.66 (0.93–2.93)

a Countries with only more than 10 cases of gestational diabetes are included to conceal the identity of women.

b Unadjusted generalized linear model.

c Generalized linear Model adjusted for age, socioeconomic position, pre-pregnancy BMI, parity and year of delivery.

## Discussion

### Key findings

This nationwide, register-based study demonstrated substantial variation in the prevalence of GDM by region and country of origin among women giving birth in Finland during a period of eleven years. After adjusting for confounders, women originating from Pakistan, Bangladesh, Sri Lanka, India, Afghanistan and Nepal (South Asia), China, Philippines, Vietnam, Thailand (East Asia), Morocco, Turkey, Iran and Iraq (the Middle East/North Africa) and the former USSR had a higher risk for GDM than Finnish origin women.

### Interpretation

Adjustment for the main risk factors of GDM, such as older age and BMI, explains some differences in the RRs in our study. The South Asian and Russian/USSR origin women were younger than Finnish origin women on average. Respectively, the Sub-Saharan origin women had a higher BMI and the East Asian and Russian/USSR origin women had a lower BMI than the Finnish origin women on average.

Although obesity is strongly associated with the risk of developing GDM, Makgoba et al. demonstrated that women with a normal BMI from African and South Asian origin had an odds ratio of 2.62 (95% CI 1.83–3.74) and 3.00 (95% CI 2.51–3.57), respectively, to develop GDM compared with the general population of women in the European countries.^26^ The difference in the prevalence of GDM increased exponentially with increasing BMI.^31^ The BMI level is not a reliable predictor of GDM in Asian women. Asian women were at a higher risk for GDM even if their BMI was below or within a normal range.^27^^,^^28^ Asian populations tend to have more visceral or central fat, which is a known risk factor for insulin resistance and cardiovascular disease.^29^ A study conducted in Norway found that among women of South Asian origin, subcutaneous fat and serum leptin levels are more likely to retain after delivery compared with women of European origin (82% were of Norwegian origin).^30^ This could increase weight retention and subcutaneous fat which may lead to excess adiposity and obesity and thereby to an increased risk of GDM in subsequent pregnancies.^30^ Another potential explanation for the higher risk of GDM among some women is genetic predisposition. A meta-analysis on genetic risk factors for GDM identified the genetic variant rs7903146 as responsible for the risk of GDM in women of Hispanic/Latina, European and Asian origin.^31^ However, future studies are warranted to further validate these findings.^31^

Two large studies from the USA have demonstrated that women who migrated from their country of birth to the USA had a higher rate of GDM compared to women of the same origin but who were born in the USA, except for women of Japanese and South Korean origin.^32^^,^^33^ However, the studies did not mention whether the difference was between the first and second generation or between the siblings. One explanation for this could be that women migrating from low- and middle-income countries to high-income countries are likely to undergo a rapid epidemiological transition due to changes in the environment and lifestyle habits. This may lead to rapid weight gain after migration.^34^ For example, there is a widespread migration of women of Asian and African origin, which traditionally have a greater proportion of women who were underweight or normal weight^27^ to countries with higher rates of overweight and obesity. After migration to the countries with higher overweight and obesity rates, changes in lifestyle and food habits may contribute to an increase in overweight and obesity rates among the migrant women placing pregnant women in these groups at a greater risk of GDM.^34^

In this study, the prevalence of GDM was about 9% among the Finnish origin, which is almost half of the current national prevalence.^21^ Our data were slightly older (i.e. from 2004 to 2014) and the national prevalence has been increasing over the years, for example, it has increased from 7% in 2008 to 19% in 2019.^21^ This is partly due to the change from risk-based to more comprehensive screening of GDM in 2008. Before 2008, pregnant women were screened for GDM if they had glucosuria, BMI ≥25 kg/m^2^, age ≥40 years, fetal macrosomia in current or previous pregnancy, GDM in a previous pregnancy, or family history of T2DM.^25^ This risk-based screening had been used in Helsinki metropolitan area hospitals before 2008, but other criteria may have been used elsewhere in Finland. Since 2008, the Current Care Guidelines recommended that GDM screening should be comprehensive, the only exception being those at very low risk.^25^ Consequently, the number of performed oral glucose tolerance tests on pregnant women doubled in Finland between 2006 and 2011.^35^ Other possible explanations for the increased prevalence of GDM are increases in the risk factor levels (e.g. the mean BMI and age of pregnant women).^21^

Our findings are in accordance with, the findings of recent studies from Denmark and Norway.^18^^,^^19^ Both studies used data from their national MBR, which are comparable to Finnish one in the data quality. Both studies used the country of birth of women to identify the country of origin whereas we used woman’s and her parent’s country of birth. Our classification of migrants’ regions of origin was based on the United Nations’ geographical classification of countries whereas the Norwegian study used seven Global Burden of Disease super regions classification.

Yuen et al. reviewed studies on the prevalence of GDM by ethnicity in 2018, including studies conducted mainly in the Australia and the USA but also one study from Italy and one from the UK.^17^ They reported a higher prevalence of GDM among migrant women of South Asian, East Asian, the Middle Eastern, African, Hispanic, Pacific Islands or indigenous Australian origin. Yet, this result should be interpreted with caution due to important differences in the categorization of geographical regions and sub-regions and different GDM diagnosis criteria in different countries.

### Strengths

This study contributed to the limited literature on GDM among migrant origin women in Finland and identified the high-risk migrant origin groups for GDM. The outcome measure (GDM) was based on standard ICD-10 codes extracted from the MBR. Furthermore, the data in the nationwide MBR have a high degree of completeness^36^ and limited risk of selection bias. The sample size was quite large in our study, and we were also able to perform several country-specific analyses for the migrant groups with a higher risk of GDM. These results are likely to be generalizable to countries outside Finland with a similar health care system.

## Limitations

As our data were based on routinely collected information, we had no information on several important migration indicators, such as migration status, length of stay, and language skills, which might have contributed to the differences between the groups. Although we have adjusted for some known confounders, we could not adjust for unhealthy diet and low physical activity which are well-known risk factors for GDM and could differ between women of migrant origin and Finnish women. Our variable on the socioeconomic position was based on employment and occupation, which was unknown for almost 30% of women from Sub-Saharan Africa and the Middle East/North Africa. Better indicators of socioeconomic position, such as the highest educational attainment and family income, should be used in future studies whenever possible.

## Suggestions for future studies and practice

Future studies could explore possible reasons for the higher GDM risk in certain migrant origin groups. Possible differences in the adverse outcomes of GDM and treatment of GDM could also be compared between the migrant origin groups and the reference group. Women originating from the high-risk countries should be offered culturally tailored care to manage GDM during pregnancy. More research is needed on how to prevent the development of GDM among women of migrant origin. The interventions carried out among pregnant women in Western countries have had limited effects on prevention of GDM.^37^ Also, they are not directly applicable to many migrant origin women due to cultural differences in dietary and physical activity habits in general and specifically, during and after pregnancy.^38^ Evidence of interventions to lose excess weight already before pregnancy is particularly warranted.

In conclusions, this study provided evidence on a higher risk of GDM among women of South Asian, East Asian and Middle Eastern/North African origin in Finland. GDM is a serious pregnancy complication and causes various short- and long-term effects on the health of both mother and the newborn. Therefore, women from these high-risk groups should be given special attention and help in the maternity care.

## Supplementary data

Supplementary data are available at *EURPUB* online.

## Funding

We would like to acknowledge the Finnish Cultural Foundation, grant number 50201295 for funding this study.

*Conflicts of interest*: None declared.

**Key points**

- Women originating from South Asian, East Asian and Middle Eastern/North African countries have a higher risk for developing GDM compared to Finnish origin women in Finland.
- When analyzed by individual country of origin, women originating from Pakistan, Bangladesh, Sri Lanka, India, Nepal, Afghanistan, China, Philippines, Vietnam, Thailand, Morocco, Turkey, Iran and Iraq and the former USSR countries had a higher risk for GDM than Finnish origin women.
- Women originating from Western Europe, Eastern Europe, Sub-Saharan African and Latin America/Caribbean countries did not differ from Finnish origin women for the risk of GDM.

## Supplementary Material

ckab078_Supplementary_Data
