# Supplementary material for: Gestational diabetes among women of migrant origin in Finland—a population-based study
Source: Eur J Public Health. 2021 May 31;31(4):784–9. doi: 10.1093/eurpub/ckab078 (PMC8561240; doi:10.1093/eurpub/ckab078)
Supplement: ckab078_Supplementary_Data [file ckab078_Supplementary_Data.docx]

eTable 1. A list of countries and numbers of women in each migrant group by country of origin (For countries with less than ten women, numbers are shown as <10 to protect the identity of the women)

| **Migrant Group** | **Number of women** |
| --- | --- |
| **Finland** | **348313** |
| **Western Europe (Western Europe/North America/Oceania)** | **2276** |
| Australia | 34 |
| Austria | 35 |
| Belgium | 17 |
| Canada | 63 |
| Cyprus | <10 |
| Denmark | 33 |
| France | 147 |
| Germany | 367 |
| Greece | 30 |
| Hungary | 211 |
| Iceland | 21 |
| Ireland | 23 |
| Italy | 103 |
| Luxembourg | <10 |
| Malta | <10 |
| New Zealand | <10 |
| Norway | 59 |
| Papua New Guinea | <10 |
| Portugal | 28 |
| Solomon Island | <10 |
| Spain | 132 |
| Sweden | 504 |
| Switzerland | 39 |
| The Netherlands (with Netherlands Antilles) | 54 |
| UK | 136 |
| USA | 230 |
| **Eastern Europe** | **2560** |
| Albania | 26 |
| Bosnia-Herzegovina | 89 |
| Bulgaria | 166 |
| Croatia | 20 |
| Czech Republic (Czechia) | 25 |
| Former Czechoslovakia | 71 |
| Former Yugoslavia | 1429 |
| Macedonia | 24 |
| Montenegro | <10 |
| Poland | 413 |
| Romania | 276 |
| Serbia | 18 |
| Slovakia | 24 |
| Slovenia | <10 |
| **Russia/former Soviet Union (USSR)** | **11 961** |
| Armenia | 17 |
| Azerbaijan | 14 |
| Belarus | 34 |
| Estonia | 3504 |
| Former USSR | 7136 |
| Georgia | <10 |
| Kazakhstan | 24 |
| Kyrgyzstan | <10 |
| Latvia | 200 |
| Lithuania | 121 |
| Moldova | 26 |
| Russia | 682 |
| Tajikistan | <10 |
| Turkmenistan | <10 |
| Ukraine | 173 |
| Uzbekistan | 15 |
| **South Asia** | **1893** |
| Afghanistan | 528 |
| Bangladesh | 257 |
| Bhutan | <10 |
| India | 623 |
| Nepal | 115 |
| Pakistan | 235 |
| Sri Lanka | 134 |
| **East Asia** | **4933** |
| Cambodia | 56 |
| China | 1131 |
| Indonesia | 89 |
| Japan | 230 |
| Laos (Lao) | 16 |
| Malaysia | 45 |
| Mongolia | <10 |
| Myanmar | 184 |
| Philippines | 477 |
| Singapore | 19 |
| South Korea | 57 |
| Thailand | 1661 |
| Vietnam | 960 |
| **Africa including Sub-Saharan Africa** | **3522** |
| Angola | 85 |
| Benin | <10 |
| Botswana | <10 |
| Burundi | <10 |
| Cameron | 107 |
| Central African Republic | <10 |
| Comoros | <10 |
| Congo | 253 |
| Cote d´Ivoire | <10 |
| Djibouti | <10 |
| Eritrea | 22 |
| Ethiopia | 208 |
| Gabon | <10 |
| Gambia | 53 |
| Ghana | 165 |
| Guinea | <10 |
| Equatorial Guinea | <10 |
| Kenya | 215 |
| Liberia | 18 |
| Madagascar | <10 |
| Malawi | <10 |
| Mauritius | <10 |
| Mozambique | <10 |
| Namibia | <10 |
| Niger | <10 |
| Nigeria | 181 |
| Rwanda | 24 |
| Seychelles | <10 |
| Senegal | 13 |
| Somalia | 1760 |
| South Africa | 23 |
| Sudan | 185 |
| Tanzania | 74 |
| Togo | <10 |
| Uganda | 30 |
| Zambia | 31 |
| Zimbabwe | <10 |
| **Middle East & North Africa** | **3440** |
| Algeria | 98 |
| Bahrain | <10 |
| Egypt | 63 |
| Iran | 554 |
| Iraq | 1264 |
| Israel | 43 |
| Jordan | 36 |
| Kuwait | 14 |
| Lebanon | 53 |
| Libya | 24 |
| Morocco | 334 |
| Oman | <10 |
| Palestine | <10 |
| Qatar | <10 |
| Saudi Arabia | 17 |
| Sierra Leone | 10 |
| Syria | 89 |
| Tunisia | 70 |
| Turkey | 755 |
| UAE | <10 |
| Yemen | <10 |
| **Latin America & Caribbean** | **736** |
| Antigua & Barbuda | <10 |
| Argentina | 44 |
| Bahamas | <10 |
| Bolivia | 22 |
| Brazil | 219 |
| Chile | 44 |
| Colombia | 58 |
| Costa Rica | <10 |
| Cuba | 43 |
| Dominican Republic | 19 |
| Ecuador | 27 |
| EL Salvador | 12 |
| Guatemala | <10 |
| Guyana | <10 |
| Haiti | <10 |
| Honduras | 12 |
| Jamaica | 13 |
| Mexico | 76 |
| Nicaragua | 10 |
| Panama | <10 |
| Paraguay | <10 |
| Peru | 76 |
| Saint Lucia | <10 |
| Samoa | <10 |
| Trinidad &Tobago | <10 |
| Uruguay | <10 |
| Venezuela | 20 |
| **Others** | **229** |
| Asylum seeker (unknown country of birth) | 201 |
| Unknown | 28 |
